# Actinic cheilitis: proposition and reproducibility of a clinical criterion

**DOI:** 10.1038/bdjopen.2017.16

**Published:** 2017-08-04

**Authors:** Nádia Antunes Poitevin, Mariana Sudati Rodrigues, Karen Loureiro Weigert, Carmen Lúcia Rodrigues Macedo, Rubem Beraldo dos Santos

**Affiliations:** 1 Dentist Private Practice, Santa Maria, Rio Grande do Sul, Brazil; 2 School of Dentristy, Pontifical Catholic University of Rio Grande do Sul, Cachoeira do Sul, Rio Grande do Sul, Brazil; 3 School of Dentistry, Lutheran University of Brazil, Cachoeira do Sul, Rio Grande do Sul, Brazil

## Abstract

**Objectives/Aims:**

The actinic cheilitis (AC) is a precancerous lip lesion seen as a consequence of chronic sun exposure. Clinically, the border between the lip’s skin and the semimucosa could be blurred; in the more aggressive cases, leucoplakia and ulcers also represent its clinical feature. It seems that no clinical criterion is universally accepted for this disease yet. Therefore, this study was carried out to make a proposition of a clinical score to actinic cheilitis (Grade I starting from dryness of vermilion to endured ulcers representing Grade IV) and to assess its reproducibility.

**Materials and Methods:**

Fifty subjects were assessed, most of whom were male, Caucasian farmers, with an average age of 46.12 (18–74) years. The obtained data were analysed by means of descriptive statistics and by Kappa test to assess the inter-examiners and the clinical Golden-Pattern concordance (95% CI).

**Results:**

During calibration, 15 patients were examined three times a week by each examiner (4) until Kappa test observed *k*=0.8 or more. In the main experiment, the inter-examiner concordance was classified between good (*k*=0.779; *P*<0.05) and very good (*k*=0.925; *P*<0.05) from the 35 examined subjects. With the Golden-Pattern, it was considered very good (*k*=0.812; *P*<0.05 to *k*=0.925; *P*<0.05).

**Discussion:**

Four examiners with different experiences could strongly suggest that after adequate calibration, it could be well applied by examiners with as much experience as a dental student.

**Conclusions:**

The authors concluded that the proposed classification was easily applied and had a very good reproducibility.

## Introduction

The actinic cheilitis (AC) is seen on the semimucosa or vermillion of the lips, mainly on the lower lip. It is related to long or intense sunlight exposure. Sometimes, AC could be defined as a ceratotic cheilitis, and it could be thought of as an early manifestation of a precancerous process. With time and without treatment, AC could become a lip’s squamous cell carcinoma (SCC).^1,2^

The biopsy demonstrated that when areas with AC are compared with normal areas of the lip, there is an altered expression of proteins such as p53 and an intensity of mast cells.^3,4^ In agreement with the histological finds, it was demonstrated that the expression of epithelial syndecan-1 was reduced when samples of normal lips were compared with the samples of AC and SCC lips.^5^ In addition, a study suggested that the expression of DNA methyltransferases plays a role in the process of carcinogenesis for AC.^6^ These facts support the thesis of AC’s malignant potential.

Although alterations in the connective tissue, such as perivascular inflammation process, elastosis and dysplasia, that can be accompanied by acanthosis, hyperplasia and hyperkeratosis or atrophy, and dysplasia in the epithelium can be seen, these histological finds are, normally, not related to the clinical features of AC.^7,8^ Atrophy, blurred demarcation between the lip’s vermilion border and the skin, and dry, scaly, swelling of the lip area are some of the several clinical possible features of AC.^2,8^

The AC prevalence can reach at least 20% based on the studies of the population of Southern Brazil.^9,10^ In addition, the potential tax of its malignant transformation is still not completely clear.^11^ As AC has a very variable clinical feature that cannot be easily related to histological finds, the present study aims to make a proposition of a clinical criterion to classify AC and to assess its reproducibility.

## Materials and Methods

This observational study was approved by the Ethics Committee of Lutheran University of Brazil (2007-391H). Each participant was informed about the objectives of the research and they gave their written consent to participate. Fifty patients with median age of 46.12 (18–74) years participated in the study; 42 of them were diagnosed with AC.

### The AC’s clinical score

The present AC’s clinical score was built starting from the descriptions made by classical and contemporaneous authors: Neville *et al.*,^2^ Silva *et al.*,^10^ Shaffer *et al.*,^12^ Regezi and Sciubba^13^ and Tommasi.^14^

**Figure 1:**
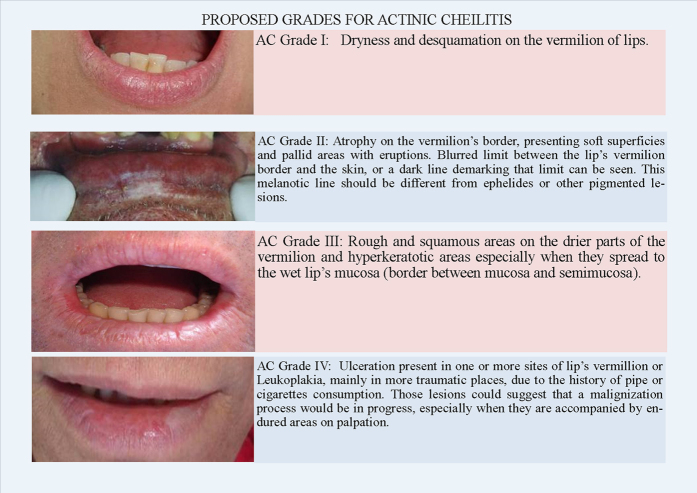
Illustration showing the four proposed grades to the AC.

The four grades are illustrated in Figure 1 and are presented as follows:

#### AC Grade I

Dryness and desquamation on the vermilion of lips.

#### AC Grade II

Atrophy on the vermilion’s border, presenting soft superficies and pallid areas with eruptions. Blurred limit between the lip’s vermilion border and the skin, or a dark line demarking that limit can be seen. This melanotic line should be different from ephelides or other pigmented lesions.

#### AC Grade III

Rough and squamous areas on the drier parts of the vermilion and hyperkeratotic areas, especially when they spread to the wet lip’s mucosa (border between mucosa and semimucosa).

#### AC Grade IV

Ulceration present in one or more sites of the lip’s vermillion or Leukoplakia, mainly in more traumatic places, due to the history of pipe or cigarettes consumption. These lesions could suggest that a malignization process would be in progress, especially when they are accompanied by endured areas on palpation.

### Statistical analysis

The obtained data were analysed by descriptive statistics and by Kappa test to evaluate the concordance between the intra-examiner, inter-examiner and the Golden-Pattern; the confidence interval was of 95%.

### Calibration of examiners applying AC’s criteria

After establishing the criteria described above, the four examiners studied and discussed it before its application. The examiners were composed of two professors of oral medicine, one student finishing dental School and one dentist with 2 years of general practice in dentistry. The team, who took note of the results, was composed of four dental students from the Scientific Initiation Program.

In this training phase, 15 (10 with and 5 without AC) patients who received treatment in the Service of Stomatology and Prevention of Oral Cancer of School of Dentistry, Lutheran University of Brazil-Campus Cachoeira do Sul were assessed. The evaluation was made 3 times once a week until the Kappa test performed by the intra- and inter-examiner reached the value of at least *k*=0.8 for all examiners.^15^

### The main experiment

In this part of the study, all exams were performed in the Gaucha’s Traditions Center from Vila Rosa, Restinga Sêca-RS, Brazil. The patient was seated on a common chair, under artificial illumination and was asked to keep his/her mouth closed, with a gentle touch between upper and lower lips. At that moment, the lip’s border between the skin and semimucosa were visually inspected. Afterwards, the whole extension of the lips vermillion was inspected as well. As the second step, the patient was asked to open his/her mouth, their lips were palpated and the Auxiliar recorded in the chart of AC’s grade attributed for that patient, without any access to the grade matched by the others examiners. It was made in an organised circuit to avoid wasting of time, and to guarantee that no examiner could know the grade attributed by the others. Therefore, each patient was assessed 4 times. At the end of all exams, the four examiners compared their results, and in 7 cases, among the 35 patients, in which there was at least one difference attributed to one or more examiners, the patients were asked to return on the following week while the examiners together decided the correct grade by assessing the first and the second examinations, this is now considered as the clinical Golden-Pattern of AC’s score.

## Results

In the present study, 35 subjects were assessed: 20 men and 15 women, 32 Caucasians, 2 mixed ethnicity and 1 Black. Among these subjects, 28 were farmers, 2 were business people, 2 were homemakers and 3 were students.

In Table 1 and in Table 2, it is possible to see the marks of each examiner as well as those of the Golden-Pattern according to the classification given to each patient.

**Figure 2:**
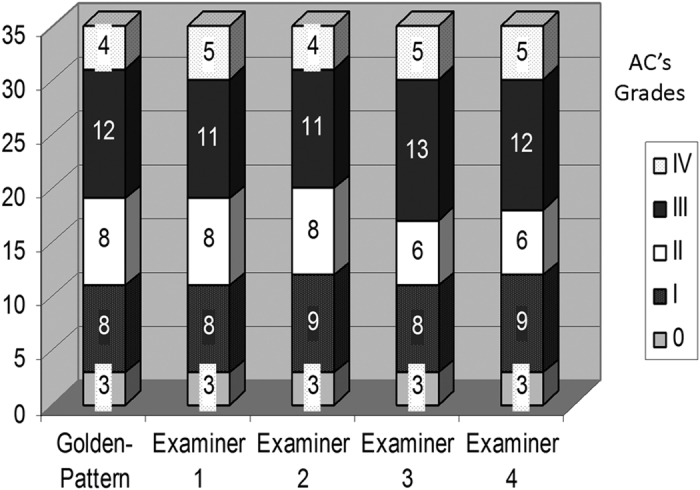
Expression of the overall results of examiners and the Golden-Pattern.

The inter-examiner concordance test was very good^15^ when the examiners were compared with the Golden-Pattern (*k*=0.81–0.925; *P*<0.05). The weaker concordance was considered good^15^ when the results of examiner 1 were compared with those of examiner 4 (*k*=0.779; *P*=0.0001) and even with those 7 patients who needed to return for another examination, *k*=0.8 yielded weaker result as well (Table 1 and Figure 2).

## Discussion

The main justification for this study was to attempt to contribute scientifically to diminish the scarce data about evaluation of the AC clinical progress. Moreover, the search for a useful tool to confidently deal with the disease in the long-term was also considered. It seems that there is not enough numbers of longitudinal studies about this theme, and the AC could sometimes be misdiagnosed as an ageing characteristic.^11^

Despite the availability of detailed descriptions in the literature,^2,8^ it seems that there is no clinically and easily accepted criterion for AC that is described widespread. Consequently, there is a background gap to support the prevention and the decision about the adequate opportunity to use or not to use a more invasive therapeutic approach to AC.

In the present study, the main sample was chosen among the members of a community of Germanic descendants from the center of the state of Rio Grande do Sul, Brazil. This specific region was chosen because most of its 800 inhabitants are Caucasians with bluish or greenish eyes. Another important factor is that most of them had their main economic activity based on tobacco and rice farming, which is commonly called outdoor labour activities. Therefore, they are within a classical group with ethnic and occupational traits that present a high risk factor for the said disease.^5,9,11^ In fact, a significant number of people had AC (46.4% of them), which made it easier to perform the study due to the abundant sample. Nevertheless, the most important aspect was to help that population to avoid lip and skin cancer with preventive and earlier diagnosis actions. This kind of cancer is a concern in Southern Brazil.^16^ It is true as beyond ethnical and occupational factors, the Middle area of the State of Rio Grande do Sul is right in the Pampa Biome, a Brazilian ecosystem where the ultraviolet rays are more aggressive and considered with extremely high radiation level throughout the year, due to the depleting Ozone layer.^17–19^

In the present study, people with ages ranging from 18 to 74 were included, so the enclosed age group would have a higher prevalence to AC, near to 40 years old, even though AC has been reported in earlier ages such as in a 16-year-old fisherman.^5,10,20^

As the clinical criterion, it seems that in the 80’s, the description of AC used to be less detailed and it was sometimes called as sealer’s or fishermen’s lip with a very strong occupational vinculation.^12^ On the other hand, contemporary authors, benefited^2,9^ by the evolution of science, gave a more detailed description that could support the foundation of our classification.

Grade I, as proposed here, is described by Silva *et al.*^10^ in a study of a community of 111 fishermen from the Island of Santa Catarina, Brazil wherein they observed that 14 of them had drier and desquamated lip vermilion, which they described as the initial feature of the disease.

Neville *et al.*,^2^ on the other hand, described the atrophy on the border of the vermillion, accompanied by soft and pale superficies with eruptions as the initial feature. This aspect was considered Grade II in our proposition. According to the same authors, with the progression of AC, a focal chronic ulceration could be seen in one or more areas of the lip, which was considered Grade IV in the present criterion.

As depicted in Figure 2, it is easy to realize that all examiners had more concordance for Grades I and IV than for Grades II and III; of course with less variation among them, the examiner’s decision becomes harder.

Considering that, four examiners with different experiences could increase the value of the proposed instrument and strongly suggest that after adequate calibration, it could be well applied by examiners with as much experience as a dental student.

In the future, with a bigger sample and longitudinal analysis, the proposed classification should be assessed better and maybe improved, given its contribution to get a better prognosis analysis to AC since the first clinical examination.

## Conclusion

The proposed criterion of AC showed itself to be of easy consumption by dental students and specialists. Furthermore, this method presented an adequate intra- and inter-examiner reproducibility.

## Figures and Tables

**Table 1:** Results matched by each examiner and of the Golden-Pattern

*Patient*	*Golden-Pattern*	*Examiner 1*	*Examiner 2*	*Examiner 3*	*Examiner 4*
1	II	II	II	II	II
2	II	II	II	II	I
3	0	0	0	0	0
4	II	II	II	II	III
5	II	III	II	III	II
6	II	II	II	II	II
7	III	III	III	III	IV
8	0	0	0	0	0
9	III	III	III	III	III
10	I	I	I	I	I
11	III	III	II	III	III
12	I	I	I	I	I
13	IV	IV	IV	IV	IV
14	III	III	III	III	III
15	I	I	I	I	I
16	III	IV	III	IV	III
17	III	III	III	III	III
18	III	II	III	III	II
19	I	I	I	I	I
20	I	I	I	I	I
21	III	III	III	III	III
22	II	II	I	II	II
23	I	I	I	I	I
24	II	II	II	II	II
25	0	0	0	0	0
26	I	I	I	I	I
27	III	III	III	III	III
28	IV	IV	IV	IV	IV
29	III	III	III	III	III
30	III	III	III	III	III
31	III	III	III	III	III
32	IV	IV	IV	IV	IV
33	II	II	II	III	III
34	I	I	I	I	I
35	IV	IV	IV	IV	IV
	Concordance with Golden-Pattern (95% CI)	Kappa (*k*) 0.888	Kappa (*k*) 0.925	Kappa (*k*) 0.887	Kappa (*k*) 0.812

Abbreviation: CI, confidence interval.

**Table 2:** Synthesis of obtained results and inter-examiners concordance

*Criterion*	*Golden-Pattern*	*Examiner 1*	*Examiner 2*	*Examiner 3*	*Examiner 4*
0	3	3	3	3	3
I	8	8	9	8	9
II	8	8	8	6	6
III	12	11	11	13	12
IV	4	5	4	5	5
Patients’	35	35	35	35	35
Concordance		with 3 *k*=0.925	*k*> 0.8	*k*> 0.8	*k*> 0.8 with examiners
Inter-examiners (95% CI)		with 4 *k*=0.779	with all others examiners	with all others examiners	2 and 3

Abbreviation: CI, confidence interval.
